# X chromosome inactivation does not necessarily determine the severity of the phenotype in Rett syndrome patients

**DOI:** 10.1038/s41598-019-48385-w

**Published:** 2019-08-19

**Authors:** Clara Xiol, Silvia Vidal, Ainhoa Pascual-Alonso, Laura Blasco, Núria Brandi, Paola Pacheco, Edgar Gerotina, Mar O’Callaghan, Mercè Pineda, Judith Armstrong, Rett Working Group, Francisco Javier Aguirre, Montserrat Aleu, Xènia Alonso, Mercè Alsius, Maria Inmaculada Amorós, Guillermo Antiñolo, Lourdes Aquino, Carmen Arellano, Gema Arriola, Rosa Arteaga, Neus Baena, Montserrat Barcos, Nuria Belzunces, Susana Boronat, Tomás Camacho, Jaume Campistol, Miguel del Campo, Andrea Campo, Ramon Cancho, Ramon Candau, Ignacio Canós, María del Carmen Carrascosa, Francisco Carratalá-Marco, Jovaní Casano, Pedro Castro, Ana Cobo, Jaime Colomer, David Conejo, Maria José Corrales, Rocío Cortés, Gabriel Cruz, Gábor Csányi, María Teresa de Santos, María de Toledo, Miguel Del Campo, Mireia Del Toro, Rosario Domingo, Anna Duat, Rosario Duque, Ana María Esparza, Rosa Fernández, Maria Carme Fons, Ana Fontalba, Enrique Galán, Pia Gallano, María José Gamundi, Pedro Luis García, María del Mar García, María García-Barcina, María Jesús Garcia-Catalan, Ángels García-Cazorla, Sixto García-Miñaur, Juan Jose Garcia-Peñas, María Teresa García-Silva, Rosa Gassio, Esther Geán, Belén Gil, Sarenur Gökben, Luis Gonzalez, Veronica Gonzalez, Julieta Gonzalez, Gloria González, Encarna Guillén, Miriam Guitart, Montserrat Guitet, Juan Manuel Gutierrez, Eva Gutiérrez, Jose Luís Herranz, Gemma Iglesias, Iva Karacic, Carlos H. Lahoz, José Ignacio Lao, Pablo Lapunzina, María Jesús Lautre-Ecenarro, María Dolores Lluch, Laura López, Asunción López-Ariztegui, Alfons Macaya, Rosario Marín, Charles M. Lourenço Marquez, Elena Martín, Beatriz Martínez, Eduardo Martínez-Salcedo, María José Mas, Gonzalo Mateo, Pilar Mendez, Amparo Morant Jimenez, Sira Moreno, Fernando Mulas, Juan Narbona, Andrés Nascimento, Manuel Nieto, Tania Fabiola Nunes, Núria Núñez, María Obón, Ignacio Onsurbe, Carlos Ignacio Ortez, Emilio Orts, Francisco Martinez, Rafael Parrilla, Samuel Ignacio Pascual, Ana Patiño, Maria Pérez-Poyato, Belén Pérez-Dueñas, Pilar Póo, Eliodoro Puche, Feliciano Ramos, Miquel Raspall, Ana Roche, Susana Roldan, Jordi Rosell, Cesar Ruiz, María Luz Ruiz-Falcó, Maria Eugenia Russi, Jordi Samarra, Victoria San Antonio, Ivan Sanchez, Xavier Sanmartin, Ana Sans, Alfredo Santacana, Sabine Scholl-Bürgi, Nuria Serrano, Mercedes Serrano, Pilar Martin-Tamayo, Adrián Tendero, Jaime Torrents, Diego Tortosa, Emma Triviño, Ledia Troncoso, Eulàlia Turón, Pilar Vázquez, Carlos Vázquez, Ramón Velázquez, Clara Ventura, Alfonso Verdú, Anna Vernet, M. Tomás Vila, Cristina Villar

**Affiliations:** 1 Molecular and Genetics Medicine Section, Hospital Sant Joan de Déu, Barcelona, Spain; 2 Facultat de Medicina, Universitat de Barcelona, Barcelona, Spain; 3 Institut de Recerca Pediàtrica, Hospital Sant Joan de Déu, Barcelona, Spain; 4 CIBER-ER (Biomedical Network Research Center for Rare Diseases), Instituto de Salud Carlos III, Madrid, Spain; 5 Neurology Service, Hospital Sant Joan de Déu, Barcelona, Spain; 6 Althaia, Manresa, Spain; 7 Balagué Center, Barcelona, Spain; 8 Catlab, Barcelona, Spain; 9 Clinical Hospital Center Zagreb, Zagreb, Croatia; 10 Complejo asistencial, Burgos, Spain; 11 Complejo Hospitalario Universitario Insular, Las Palmas de Gran Canaria, Spain; 12 Consorci Sanitari, Terrassa, Spain; 13 Hospital de Fuenlabrada, Madrid, Spain; 14 Hospital Central Asturias, Asturias, Spain; 15 Hospital Francesc De Borja, Valencia, Spain; 16 Hospital General de Granollers, Barcelona, Spain; 17 Hospital Universitario Río Hortega, Valladolid, Spain; 18 Hospital General de Vic, Barcelona, Spain; 19 Hospital General Mancha Centro, Ciudad Real, Spain; 20 Hospital General Universitario de Albacete, Albacete, Spain; 21 Hospital General Universitario de Alicante, Alicante, Spain; 22 Hospital Universitario de Getafe, Getafe, Spain; 23 Hospital Universitario de Guadalajara, Guadalajara, Spain; 24 Hospital Universitario de Valme, Sevilla, Spain; 25 Hospital Virgen de la Luz, Cuenca, Spain; 26 Hospital Virgen de la Salud, Toledo, Spain; 27 Hospital Virgen del Camino, Pamplona, Spain; 28 Laboratorio Echevarne, Barcelona, Spain; 29 Lema & Bandin Laboratorios, Vigo, Spain; 30 Medizinische Universität Innsbruck, Innsbruck, Austria; 31 Reference Laboratory, Barcelona, Spain; 32 Hospital de la Vall d’Hebrón, Barcelona, Spain; 33 Centro privado, Valencia, Spain; 34 Hospital Infantil Universitario Niño Jesús, Madrid, Spain; 35 Hospital de Sabadell, Barcelona, Spain; 36 Hospital La Paz, Madrid, Spain; 37 Hospital Materno-Infantil de Badajoz, Badajoz, Spain; 38 Hospital Infantil de La Arrixaca, Murcia, Spain; 39 Hospital Universitario Puerta del Mar, Cádiz, Spain; 40 Hospital San Borja Arriaran, Santiago, Chile; 41 Hospital Clínico San Carlos, Madrid, Spain; 42 Hospital Universitario General de Castellón, Castellón, Spain; 43 Hospital de Mataró, Mataró, Spain; 44 Hospital de Navarra, Pamplona, Spain; 45 Hospital de la Santa Creu i Sant Pau, Barcelona, Spain; 46 Hospital Gregorio Marañón, Madrid, Spain; 47 Hospital Joan XXIII, Tarragona, Spain; 48 Hospital Son Dureta, Palma de Mallorca, Palma, Spain; 49 Hospital Torrecardenas, Almería, Spain; 50 Hospital Universitari Dr. Josep Trueta, Girona, Spain; 51 Hospital Universitari San Juan, Alicante, Spain; 52 Hospital Universitario 12 de Octubre, Madrid, Spain; 53 Hospital Universitario Doctor Peset, Valencia, Spain; 54 Hospital Universitario Marqués de Valdecilla, Santander, Spain; 55 Hospital Universitario Nuestra Señora de la Candelaria, Santa Cruz de Tenerife, Spain; 56 Hospital Universitario Reina Sofía, Córdoba, Spain; 57 Hospital Universitario Severo Ochoa, Madrid, Spain; 58 Hospital Universitario Virgen de las Nieves, Granada, Spain; 59 Hospital Universitario Virgen del Rocio, Sevilla, Spain; 60 Hospital Universitario Virgen Macarena, Sevilla, Spain; 61 Complejo Hospitalario de Jaén, Jaén, Spain; 62 Ege Ünŭversŭtesŭ Tip Fakültesŭ Pedŭatrŭ AD, Ŭzmŭr, Turkey; 63 Consorcio Hospital General Universitario de Valencia, Valencia, Spain; 64 Instituto Valenciano de Neurociencias, Valencia, Spain; 65 Clínica Universitaria de Pamplona, Pamplona, Spain; 66 Medical Genetics Service, Clinics Hospital of Ribeirão Preto, University of São Paulo, São Paulo, Brazil; 67 Hospital Clínico Universitario de Valladolid, Valladolid, Spain; 68 Hospital Clínico Universitario Lozano Blesa, Zaragoza, Spain; 69 Hospital Punta Europa, Cádiz, Spain; 70 Hospital Costa del Sol, Málaga, Spain; 71 Hospital de Basurto, Bilbao, Spain; 72 Hospital de Cruces, Bilbao, Spain; 73 Hospital de Donostia, San Sebastián, Spain; 74 Hospital Cormarcal de Figueres, Girona, Spain

**Keywords:** Genetics, Molecular medicine

## Abstract

Rett syndrome (RTT) is a severe neurological disorder usually caused by mutations in the *MECP2* gene. Since the *MECP2* gene is located on the X chromosome, X chromosome inactivation (XCI) could play a role in the wide range of phenotypic variation of RTT patients; however, classical methylation-based protocols to evaluate XCI could not determine whether the preferentially inactivated X chromosome carried the mutant or the wild-type allele. Therefore, we developed an allele-specific methylation-based assay to evaluate methylation at the loci of several recurrent *MECP2* mutations. We analyzed the XCI patterns in the blood of 174 RTT patients, but we did not find a clear correlation between XCI and the clinical presentation. We also compared XCI in blood and brain cortex samples of two patients and found differences between XCI patterns in these tissues. However, RTT mainly being a neurological disease complicates the establishment of a correlation between the XCI in blood and the clinical presentation of the patients. Furthermore, we analyzed *MECP2* transcript levels and found differences from the expected levels according to XCI. Many factors other than XCI could affect the RTT phenotype, which in combination could influence the clinical presentation of RTT patients to a greater extent than slight variations in the XCI pattern.

## Introduction

Rett syndrome (RTT, OMIM #312750) is a severe neurodevelopmental disorder characterized by a period of normal development until 6-18 months of age followed by a regression of neurological traits. RTT features include compromised brain functions, severe mental retardation, epilepsy, regression of purposeful hand use and language, breathing disturbances, gait apraxia and repetitive stereotyped hand movements^1–3^. RTT has an incidence of 1:10,000–20,000 live births and affects mainly young females^4^, being the second most common cause of severe mental retardation in females after Down syndrome.

The association of RTT with mutations in methyl-CpG binding protein 2 (*MECP2*; Xq28; OMIM *300005) gene was recognized in 1999^2^. Since then, more than 800 different mutations in *MECP2* have been identified in more than 95% of patients with classic RTT^5,6^. There are also some atypical RTT variants, such as the early onset seizure variant and the congenital variant, which have been associated with mutations in cyclin-dependent kinase-like 5 (*CDKL5*; Xp22; OMIM *300203) and forkhead box protein G1 (*FOXG1*; 14q12; OMIM *164874), respectively^7,8^. However, the vast majority of RTT patients have a *de novo* mutation in *MECP2*, and there are 8 mutation hotpots with recurrent mutations (p.Thr158Met, p.Arg255*, p.Arg168*, p.Arg306Cys, p.Arg294*, p.Arg270*, p.Arg133Cys and p.Arg106Trp), which are responsible for over 60% of all RTT cases^9,10^.

Increasing experience has shown that RTT patients present a large degree of phenotypic variation^2^. Patients with truncating mutations in *MECP2* tend to show a more severe phenotype than those with missense mutations^4^, and there are also phenotypical presentation differences between patients with the same mutation^11–13^.

These clinical differences have been attributed, at least in part, to X chromosome inactivation (XCI). Through the XCI process, mammalian female cells inactivate one of the two X chromosomes to compensate for gene dosage. XCI is a stochastic process that takes place in the initial stages of the embryogenesis, causing a mosaic expression of X-linked genes in the adult organism^3,14,15^. Since *MECP2* is located on the X chromosome, the severity of RTT could be theoretically regulated by XCI, showing a more severe phenotype as more cells express the mutated *MECP2*^14^.

Some cases of healthy carriers of RTT-causing mutations with highly skewed XCI patterns have been documented^14,16,17^, as have cases of RTT patients with milder symptoms who also presented a skewed XCI pattern^13,17,18^. However, in most XCI studies in RTT, the phase of the two X chromosomes was not determined, so the XCI pattern could only be classified as either skewed or random. Therefore, no evidence of whether the preferentially inactivated chromosome was the mutant or the wild-type (WT) could be obtained.

We have developed an allele-specific methylation-based assay to evaluate methylation on the loci of several recurrent *MECP2* mutations, allowing for evaluation of the XCI pattern while taking into account which is the mutant and which is the wild-type allele. We compared the results from the classical androgen receptor assay for evaluating X chromosome inactivation (XCI-AR) with the allele-specific X chromosome inactivation (XCI-AS) assay we developed. We also compared all XCI results with a score of clinical severity of the clinical presentation of RTT to determine if we could correlate the XCI pattern with milder or more severe forms of RTT. Our cohort included 221 RTT patients with several recurrent mutations and two deletions in *MECP2*, for whom we could evaluate XCI patterns in blood. Moreover, we also assessed XCI in brain samples of two patients and compared the XCI status to blood to determine if it could be used as an accurate predictor. Finally, we measured *MECP2* RNA levels in brain samples to determine whether they correlated with the XCI pattern detected.

## Results

### Allele-specific X chromosome inactivation and XCI skewing in blood samples

For each patient, we performed an XCI-AR and the corresponding XCI-AS when blood samples were available (174/221 patients), and we also calculated the global score of the clinical presentation when clinical data were available (181/221 patients). The reference values for considering an XCI pattern as skewed in the literature are usually established at an 80:20 ratio^14,19^, so we also used that threshold to allow the comparison of our results with previous studies. The entire list of XCI results and clinical scores for all patients can be found in Supplementary Table S1.

The overall tendency of our cohort was to have random XCI. However, 9.8% of our patients showed a skewed XCI pattern (80:20 or higher; Table 1), which is similar to what was found in other studies^13,20^. No patients with p. R152R, p.T158M or p. P225R mutations showed skewed XCI patterns in either XCI-AR or XCI-AS.

**Table 1:** Proportion of patients per mutation with a skewed XCI pattern according to at least one of the two techniques used for assessing XCI (XCI-AR and XCI-AS).

Mutation	Type of mutation	MeCP2 region	Number of patients with skewed XCI	% of patients with skewed XCI
c.455C > G (p.P152R)	Missense	MBD	0/6	0%
c.473C > T (p.T158M)	Missense	MBD	0/33	0%
c.502C > T (p.R168X)	Nonsense	IDR	5/29	17.2%
c.674C > G (p.P255R)	Missense	TRD	0/2	0%
c.763C > T (p.R255X)	Nonsense	TRD	4/36	11.1%
c.806delG (p.G269fs)	Frameshift	TRD-NLS	1/11	9.1%
c.808C > T (p.R270X)	Nonsense	TRD-NLS	4/20	20%
c.880C > T (p.R294X)	Nonsense	TRD	1/20	5%
c.916C > T (p.R306C)	Missense	TRD	1/15	6.7%
Large deletions	Deletion	Exons 3-4	1/2	50%
All	—	—	17/174	9.8%

When we applied the 80:20 skewing threshold, 17 out of 174 patients presented a skewed XCI pattern according to at least one of the two XCI assays performed (Table 2). We compared these patients’ clinical severity scores with the average clinical score of RTT patients with the same mutation. We found that, when the clinical score was available, in the majority of cases this value was included in the interval of µ ± σ (central 68% of individuals in a normal distribution) of the patients with the same mutation.

**Table 2:** Data of patients with skewed XCI according to at least one of the two assays.

Patient Number	XCI-AR	XCI-AS		Global Score
		WT	Mut	
**Patients with c.502C** > T (p.Arg168*) mutation			$\bar{{\rm{X}}}$ = 13.12 (SD = 3.361)	
P47	n.i.	81.5	18.5	13
P60	84:16	28	72	16
P68	75:25	15.5	84.5	NA
P70	85:15	35	65	NA
P74	81:19	55.5	44.5	NA
**Patients with c.763C** > T (p.Arg255*) mutation			$\bar{{\rm{X}}}$ = 15.21 (SD = 3.213)	
P83	85:15	57	43	NA
P84	87:13	55.5	44.5	13
P85	80:20	28	72	14
P107	87:13	68	32	11
**Patients with c.806delG (p.Gly269fs) mutation**			$\bar{{\rm{X}}}$ = 14.29 (SD = 4.112)	
P139	82:18	58	42	NA
**Patients with c.808C** > T (p.Arg270*) mutation			$\bar{{\rm{X}}}$ = 14.69 (SD = 3.846)	
P143	97:3	16	84	18
P144	84:16	21	79	NA
P145	81:19	30	70	9
P146	80:20	73	27	13
**Patients with c.880C** > T (p.Arg255*) mutation			$\bar{{\rm{X}}}$ = 10.46 (SD = 2.993)	
P191	89:11	49	51	NA
**Patients with c.916C** > **T (p.Arg306Cys) mutation**			$\bar{{\rm{X}}}$ = 11.18 (SD = 3.065)	
P195	89:11	59.5	40.5	9
**Patients with deletions in** ***MECP2***				
P220	88:12	6.73	93.27	NA

The XCI-AR column shows the results of the AR XCI assay (percentage of inactivation of each allele). The XCI-AS WT and Mut columns show the results of the allele-specific XCI assay (percentage of inactivation of each allele, mean of two replicates n = 2 or three replicates n = 3 in the cases of the deletions). The Global Score column shows the average ($\bar{{\rm{X}}}$) score and its standard deviation (SD) in brackets for the patients of our cohort with each mutation. Bold formatting indicates patients with a clinical score lower than the interval µ-σ for the average clinical score of their mutation. n.i. = polymorphism noninformative for the assay. NA = clinical data not available.

There were only two patients who had a clinical score lower than the interval µ-σ for their mutation (P107 and P145, Table 2, in bold). In the case of patient P107, the preferentially inactivated allele was the WT allele, while in the case of patient P145 the mutant allele was inactivated. The results from patient P145 seem to be consistent with the theory that when the chromosome that harbors the *MECP2* mutation is preferentially inactivated, the clinical presentation of RTT may be milder.

### Allele-specific X chromosome inactivation and XCI skewing in brain samples

We also performed XCI-AR and XCI-AS assays in samples of several brain regions of two patients with the c.763C > T mutation (Table 3). The XCI-AS assay was useful for assessing the XCI pattern in both patients, but especially in patient P119, since in this case, the polymorphism in the AR locus was noninformative for the XCI-AR assay.

**Table 3:** Data of patients P109 and P119 with the c.763C > T mutation.

Sample	XCI-AR	XCI-AS	
		WT	Mut
**Patient 109** (**Clinical score** = **20**)			
Frontal Cortex	65:35	26	74
Occipital Cortex	58:42	59	41
Parietal Cortex	64:36	40	60
Temporal Cortex	60:40	32	68
White matter	59:41	23	77
Brain stem	59:41	31	69
Striatum	61:39	51	49
Cerebellum	55:45	43	57
Blood	73:27	64	36
**Patient 119** (**Clinical score** = **19**)			
Frontal Cortex	n.i.	48	52
Occipital Cortex	n.i.	NA	NA
Parietal Cortex	n.i.	56	44
Temporal Cortex	n.i.	73	27
White matter	n.i.	46	54
Brain stem	n.i.	38	62
Striatum	n.i.	50	50
Cerebellum	n.i.	50	50
Blood	n.i.	34	66

The XCI-AR column shows the results of the AR XCI assay (percentage of inactivation of each allele). The XCI-AS WT and Mut columns show the results of the allele-specific XCI assay (percentage of inactivation of each allele). n.i. = polymorphism noninformative for the assay. NA = data not available.

Although no samples showed skewed XCI by either assay, there was no clear homogeneity among blood and brain samples. Some samples, such as the frontal cortex or the white matter sample of patient P109, showed an XCI pattern closer to the skewing threshold than other regions, such as the cerebellum, of the same patient. In patient P119, the vast majority of samples were close to the random XCI pattern, but the temporal cortex sample showed an XCI pattern closer to the skewing threshold.

### Brain RNA analysis

**Figure 1:**
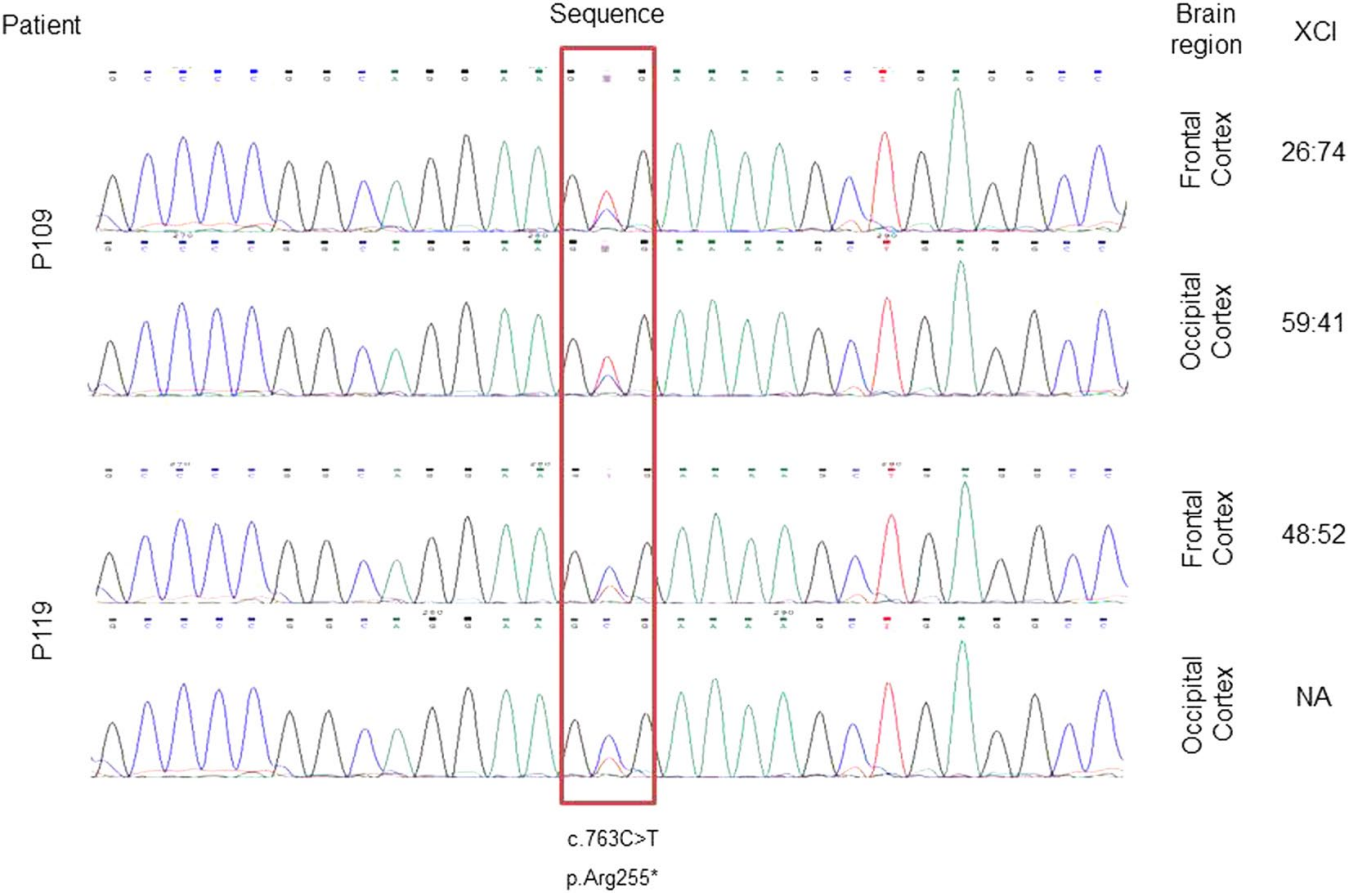
Brain RNA Sanger Sequencing. cDNA analysis of brain samples. Electropherograms obtained from Sanger sequencing of frontal and occipital cortex cDNA samples. Blue peaks correspond to the C allele (WT), while red peaks correspond to the T allele (mutated), and the red box highlights the locus of the c.763C > T mutation in heterozygosis. Inactivation ratios are shown as inactivation WT:inactivation Mut.

Finally, we analyzed frontal and occipital cortex RNA samples. We performed RT-PCR to obtain cDNA samples so that we could perform Sanger sequencing to check if we could detect the presence of one allele over the other (Fig. 1).

In cDNA samples from patient P109, the T allele (mutated allele) was overrepresented, while in samples from patient P119, the C allele (WT allele) was overrepresented. However, both patients presented a severe form of RTT, with clinical scores of 20 and 19, respectively.

The cDNA analysis was not conclusive since Sanger sequencing is not the best technique for quantifying the RNA of each allele. However, the sequencing analysis seemed to indicate that one allele was more frequently present than the other, although the XCI assay results showed inactivation patterns that did not reach the threshold for classifying the XCI pattern as skewed in any of the two patients and regions.

**Figure 2:**
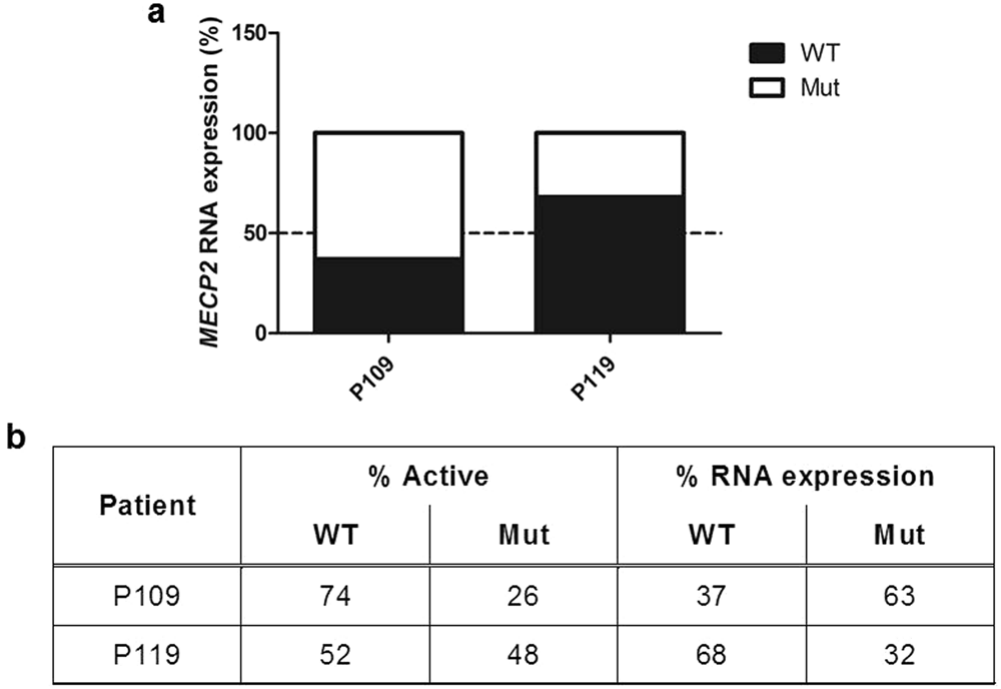
Brain RNA qRT-PCR analysis and comparison with XCI-AS assay results. (**a**) cDNA analysis of brain samples. The results obtained by qRT-PCR of frontal cortex RNA samples (% of expression of each allele). The discontinuous line indicates 50% of the expression of each allele (each allele is equally present in the sample). (**b**) Comparison of XCI and qRT-PCR data from patients P109 and P119 with the c.763C > T mutation. Data are shown as % of activation of each X chromosome (% Active) and % RNA expression measured by qRT-PCR.

We later confirmed our findings in the frontal cortex samples by qRT-PCR, a more suitable technique for quantifying RNA levels (Fig. 2a,b). We found that in samples from patient P109, the mutated allele was overexpressed, while in samples from patients P119, the WT allele was overexpressed.

## Discussion

The XCI-AS assay allowed us to describe the XCI patterns of patients previously classified as noninformative by the classical XCI-AR assay and to identify which *MECP2* allele (mutated or WT) was preferentially inactivated in cases of skewed XCI pattern.

Differences between the XCI patterns obtained by both techniques can be explained because in each technique, the methylation status is only analyzed at a single locus, and the methylation of a single cytosine residue may not be representative of the inactivation status of the entire X chromosome^21,22^. Different studies have shown that when methylation in several loci of the X chromosome is assessed, different ratios of XCI can be obtained, with up to 27% of variation^21,22^. Therefore, the use of several loci for characterizing XCI would indicate the true XCI pattern more consistently^21^.

Gathering data from both XCI assays performed with samples of 174 patients, we found that 9.8% of patients had skewed XCI patterns (80:20 XCI ratio or higher). Other studies have found either similar results^13,20^ or a considerably higher incidence of skewing, up to 43%, among RTT patients^23^. Some authors claim that most of the patients who meet the diagnostic criteria for RTT have a random XCI pattern, while those with skewed XCI patterns may not meet all the criteria and therefore are not included in some RTT studies^18^.

However, the percentage of patients in our cohort with skewed XCI patterns varied among different types of mutations. Mutations that produce a truncated protein result in a more severe phenotype than missense mutations^23^, and skewed XCI patterns were more common in RTT patients with deletions and nonsense mutations than in those with missense mutations. This could be due to a protective effect related to the severity of the mutation. It is possible that mutations producing a less functional, truncated protein (deletions and nonsense mutations) cause cells to preferentially inactivate the X chromosome harboring the mutation. It has been shown that skewed XCI can be caused by a selective advantage of cells with a particular active X chromosome proliferating faster than cells where the other X chromosome is active^15,24,25^. This type of skewing has been described in up to 50% of familial cases of X-linked mental retardation disorders^26^.

This skewed proliferation could be the case for patient P220 (Table 2), who had a large deletion in *MECP2* and showed a skewed XCI pattern (88:12) by the XCI-AR assay. In this patient, the XCI-AS assay confirmed an extremely skewed XCI pattern and that the preferentially inactivated allele was the mutated allele at a ratio of 93:7. We also found this tendency in several patients with p.Arg168* (P60, P68, P70; Table 2) p.Arg255* (P85; Table 2) and p.Arg270* (P143, P144, P145; Table 2) mutations. However, there were other patients with these same mutations with skewed XCI according to the XCI-AR assay who showed a preferential inactivation of the WT allele when the XCI-AS assay was performed, such as P146 (Table 2). Patient P47 (Table 2), who was noninformative for the XCI-AR assay, also showed a preferential inactivation of the WT allele at a ratio of 81:19 when the XCI-AS assay was performed. These last patients do not support the abovementioned hypothesis.

We found no substantial correlation between the XCI patterns in blood and the clinical presentation of RTT following the scale of evaluation of the RTT phenotype by Monrós, *et al*.^27^ (data not shown). We did not observe consistent increases or decreases in the clinical score of RTT patients with a preferential inactivation of the WT or mutated alleles in blood samples.

It has been published that XCI patterns can vary among different tissues^22,28^. Indeed, we compared the XCI patterns of blood and brain samples of the same patient, and they did not show homogeneous XCI patterns. Although they were small, there was also a slight difference in the XCI patterns between different brain regions of the same patient.

Moreover, it has been shown that blood is especially prone to XCI skewing^29^ because of the proliferation of different clones of lymphocytes under different conditions^22,29^. In fact, blood XCI patterns have shown variations at different time points in different studies^14^. For two of the patients included in the study (P9 and P199; Table S5), we compared two different blood samples from two different extractions. Both patients showed some differences in the results of the XCI assays in the two extraction samples.

The lack of a direct correlation between the XCI patterns in blood and the clinical presentation of RTT could be explained by different reasons. First, we observed that the XCI patterns in blood and different regions of the brain are not necessarily homogeneous. Therefore, if RTT symptoms are caused mainly by the lack of *MECP2* function in the brain, it is expected that the severity of the phenotype will be more related to the XCI pattern in the brain than to the XCI pattern in the blood.

Moreover, there are many other factors that can influence the presentation of the RTT phenotype, such as other polymorphisms and genetic variants, the expression levels of other genes and environmental conditions^4^. It is likely that the combination and addition of these additional factors can influence the phenotype to a greater extent than only the XCI pattern in the brain.

RTT symptoms arise from either a partial or a complete loss of function of *MECP2* in neurons^13,30^. RTT affects mainly females, partly because a complete loss of function of *MECP2* in males is so damaging that it can cause death in the first months of life or even before birth. The severity of the male phenotype points towards a dose-dependent mechanism of action of *MECP2*, where the expression of the mutant *MECP2* in a high proportion of cells causes the RTT phenotype^13,31^. It is possible that in females, slight deviations from random 50:50 XCI ratios do not cause sufficient changes in the levels of the mutant *MECP2* in the brain to be translated into a different phenotype.

However, it is possible that in more extreme cases, the effect is more remarkable. This could be similar to the case of female carriers of the *MECP2* duplication who show an extremely skewed XCI pattern with the mutant chromosome inactivated in most of their cells. In these cases, where a greater number of cells have inactivated the mutant chromosome, the effects of the XCI pattern are more important and cause the carrier of the *MECP2* duplication not to present the *MECP2* duplication syndrome. The same phenomenon could occur with pathogenic mutations in *MECP2*. If there is an extremely skewed XCI pattern in the brain, where a greater number of cells express the WT copy of *MECP2*, a threshold of *MECP2* function could be reached, and the RTT phenotype would therefore not be expressed. In some familial cases of RTT, it has been observed that a healthy mother with extremely skewed XCI can be a carrier of a pathogenic mutation responsible for causing RTT in her offspring^16–18^, although she remains asymptomatic. Some authors have claimed that these familial cases of RTT are only possible due to the presence of two coincident traits: RTT and the trait for skewed XCI, which would be genetically determined^14,16^.

The differences between the XCI patterns measured and the levels of each allele observed in Sanger sequencing and qRT-PCR could be due to RNA degradation, both in the postmortem interval and during life due to the nonsense-mediated mRNA decay (NMD) pathway, which could degrade mutant mRNA because of its potential to be translated into a truncated protein.

However, brain RNA levels of each allele seemed to show discrepancies with the XCI patterns identified in our XCI assays. Some authors have noticed discordances between the XCI pattern according to the XCI-AR assay and the quantification of the AR gene expression^32^. These discrepancies suggest, first, that the methylation assay may not always be representative of XCI and, second, that gene transcript levels may be regulated by more factors than XCI.

The difference between the XCI pattern and the final RNA levels of each allele suggests that the levels of *MECP2* are not directly determined by the XCI pattern and that there could be mechanisms other than XCI involved in regulating *MECP2* transcript levels. Consistent with what we have discussed, there might be other genes involved in regulating *MECP2* transcription and/or RNA degradation, causing changes in the final levels of functional *MECP2*^10^. Therefore, XCI may not necessarily be determining the severity of the clinical presentation of RTT, which would be more related to the levels of functional *MECP2* in the brain^30,31^.

Nevertheless, it is important to keep in mind that we are measuring *MECP2* transcript levels from brain bulk RNA. Since different neuronal types have showed diverse transcriptional profiles in several studies^33^, the levels of the *MECP2* transcripts we measured do not necessarily reflect these transcript’s levels in neurons relevant for RTT pathophysiology.

Although one patient showed higher levels of the *MECP2* mutant transcript than the other, the clinical severity scores of both patients were not dissimilar (20 vs 19). This score similarity supports the hypothesis that slight deviations from a 1:1 ratio of each allele produce little to no change in the RTT phenotype. It is possible that more consistent differences would be noticeable if one allele was more prevalent than the other, such as in asymptomatic carriers with an XCI pattern close to the 100:0 ratio.

In conclusion, our results show that the relationship between XCI and the severity of the RTT phenotype is not straightforward. Factors other than XCI can influence *MECP2* transcript levels, and presumably many additional factors, such as genetic polymorphisms and the expression of other genes, may influence the final clinical presentation of RTT. Therefore, probably only extremely skewed XCI patterns affecting neurons can be correlated with milder forms of RTT or asymptomatic carriers.

## Materials and Methods

### Sample material

The study cohort consisted of 221 RTT patients with one of the 9 following recurrent mutations in the *MECP2* gene: c.455C > G-p.Pro152Arg (6 patients), c.473C > T-p.Thr158Met (36 patients), c.502C > T-p.Arg168* (38 patients), c.674C > G-p.Pro225Arg (2 patients), c.763C > T-p.Arg255* (47 patients), c.806delG-p.G269fs (13 patients), c.808C > T-p.Arg270* (31 patients), c.880C > T-p.Arg294* (21 patients) and c.916C > T-p.Arg306Cys (25 patients); and 2 patients with a large deletion in *MECP2*.

Samples of blood genomic DNA (gDNA) were obtained from peripheral blood leukocytes. Samples of brain gDNA were obtained postmortem from several brain regions (frontal, occipital, temporal and parietal cortex; white matter, brain stem, striatum and cerebellum) of two patients with c.763C > T mutation. RNA was also obtained from the frontal and occipital cortices of such patients. DNA samples were isolated using the saline extraction kit PUREGENE® DNA Isolation Kit of Gentra Systems®, and brain RNA samples were extracted using TRIzol™ Reagent from Invitrogen™.

### Ethical approval and informed consent

The study was approved by the ethical committees of Hospital Sant Joan de Déu, CEIC: Comitè d’Ètica d’Investigació Clínica- Fundació Sant Joan de Déu (internal code: PIC-101-15). Written informed consent from the legal guardians of the patients was obtained in accordance with the corresponding ethical protocols to perform the genetic studies, and tissue samples from patients and controls were obtained according to the Helsinki Declaration of 1964, as revised in 2001^34^.

### HpaII and HinfI digestion

Digestion of gDNA samples was performed with one of the methylation-sensitive restriction enzymes *HpaII* or *HinfI* (New England BioLabs® Inc.), depending on the presence of the relevant enzyme target sequences near the studied loci. In the AR, c.455C > G, c.473C > T, c.502C > T, c.674C > G, c.763C > T, c.806delG, c.808C > T, c.880C > T, c.916C > T and deletion 2 (NM_004992.3: c.887_10015 + 18460del) loci assays *HpaII* was used, while in the deletion 1 (NM_004992.3: c.27-10677_1192del) locus assay *HinfI* was used. A total volume of 500 ng of gDNA was digested with 0.5 μL of enzyme in a 25 μL reaction volume in CutSmart 1x Buffer (New England Biolabs® Inc.). Digestions were incubated at 37 °C for 20 minutes followed by another 20 minutes at 80 °C for enzyme inactivation, as established in the enzyme protocol.

### PCR amplification and fragment analysis

A pair of primers with the sequences described in Allen, *et al*.^35^ was used to amplify the AR polymorphic locus. Allele-specific primers were designed for each *MECP2* recurrent mutation included in the study. Primer design was carried out following the recommendations in Liu, *et al*.^36^. For the deletion assays, a forward primer was designed inside the deletion locus and another primer immediately after the deletion; they were both amplified with a reverse primer outside the deleted region. All primers used were designed using Primer3web version 4.1.0^37,38^, and they are shown together with PCR conditions for each pair in Supplementary Tables S2, S3 and S4. One primer of each pair was FAM-labeled at the 5′ end.

PCR amplification was performed using the resulting DNA after the digestion and nondigestion of each sample. PCR products were analyzed on a 3500 Genetic Analyzer (Applied Biosystems®) using GeneScan™ – 500 LIZ® Size Standard of Applied Biosystems® as an internal size standard and Peak Scanner Software v1.0. The X chromosome inactivation ratios were calculated as described elsewhere^35^.

### Brain RNA analysis

RT-PCR was performed with frontal and occipital cortex RNA of two patients with the c.763C > T mutation, following the recommendations provided with the SuperScript™ III First-Strand Synthesis SuperMix for qRT-PCR from Invitrogen™. Subsequently, Sanger sequencing of the cDNA obtained in the RT-PCR reaction was performed. qPCR was performed in a QuantStudio™ 6 Flex Real-Time PCR System (Applied Biosystems™) with TaqMan™ Gene Expression Master Mix (Applied Biosystems™) and specific TaqMan™ MGB probes to amplify the mutant and the wild-type alleles. qPCR data were analyzed using the comparative Ct method. Primers and probes were designed using Primer3web version 4.1.036,37, and they are listed in Supplementary Table S5.

### Patient phenotype evaluation and correlation analysis

When clinical data were available (181/221 patients), the RTT phenotype was evaluated, and a score was assigned following the scale of evaluation of the RTT phenotype published by Monrós, *et al*.^27^.

The linear correlation between the inactivation patterns of the WT allele and the global score of each patient was evaluated using statistical methods that are based on Ordinary Least Squares (OLS) regression models, grouping patients with the same mutation.

## Supplementary information

X chromosome inactivation does not necessarily determine the severity of the phenotype in Rett syndrome patients

## Data Availability

All data from this article is available in the Supplementary Data.

## References

[1] Neul JL (2010). Rett syndrome: Revised diagnostic criteria and nomenclature. Ann. Neurol..

[2] Weaving LS, Ellaway CJ, Gécz J, Christodoulou J (2005). Rett syndrome: clinical review and genetic update. J Med Genet.

[3] Ip JPK, Mellios N, Sur M (2018). Rett syndrome: Insights into genetic, molecular and circuit mechanisms. Nat. Rev. Neurosci..

[4] Liyanage VRB, Rastegar M (2014). Rett syndrome and MeCP2. NeuroMolecular Med..

[5] Landucci E (2018). iPSC-derived neurons profiling reveals GABAergic circuit disruption and acetylated α-tubulin defect which improves after iHDAC6 treatment in Rett syndrome. Exp. Cell Res..

[6] Vidal S (2017). The utility of Next Generation Sequencing for molecular diagnostics in Rett syndrome. Sci. Rep..

[7] Weaving LS (2004). Mutations of CDKL5 cause a severe neurodevelopmental disorder with infantile spasms and mental retardation. Am. J. Hum. Genet..

[8] Mencarelli MA (2010). Novel FOXG1 mutations associated with the congenital variant of Rett syndrome. J. Med. Genet..

[9] Percy AK (2010). Rett syndrome diagnostic criteria: Lessons from the Natural History Study. Ann. Neurol..

[10] Ehrhart F (2016). Rett syndrome - Biological pathways leading from MECP2 to disorder phenotypes. Orphanet J. Rare Dis..

[11] Gonzales ML, LaSalle JM (2010). The role of MeCP2 in brain development and neurodevelopmental disorders. Curr. Psychiatry Rep..

[12] Hoffbuhr KC, Moses LM, Jerdonek MA, Naidu S, Hoffman EP (2002). Associations between MeCP2 mutations, X-chromosome inactivation, and phenotype. Ment. Retard. Dev. Disabil. Res. Rev..

[13] Amir RE (2000). Influence of mutation type and X chromosome inactivation on Rett syndrome phenotypes. Ann. Neurol..

[14] Vacca M, Della Ragione F, Scalabrì F, D’Esposito M (2016). X inactivation and reactivation in X-linked diseases. Semin. Cell Dev. Biol..

[15] Gartler, S. M. & Goldman, M. A. X-Chromosome Inactivation. *Encycl*. *Life Sci*. 1–6, 10.1038/npg.els.0004172 (2001).

[16] Sirianni N, Naidu S, Pereira J, Pillotto RF, Hoffman EP (1998). Ret Syndrome: Confirmation of X-Linked Dominant Inheritance, and Localization of the Gene to Xq28. Am. J. Hum. Genet.

[17] Zhang Q (2017). Familial cases and male cases with MECP2 mutations. Am. J. Med. Genet. Part B Neuropsychiatr. Genet..

[18] Wan M (1999). Rett Syndrome and Beyond: Recurrent Spontaneous and Familial MECP2 Mutations at CpG Hotspots. Am. J. Hum. Genet..

[19] Ørstavik KH (2009). X chromosome inactivation in clinical practice. Hum. Genet..

[20] Van Den Veyver IB, Zoghbi HY (2001). Mutations in the gene encoding methyl-CpG-binding protein 2 cause Rett syndrome. Brain Dev..

[21] Bertelsen B, Tümer Z, Ravn K (2011). Three new loci for determining X chromosome inactivation patterns. J. Mol. Diagnostics.

[22] De Hoon B, Monkhorst K, Riegman P, Laven JSE, Gribnau J (2015). Buccal swab as a reliable predictor for X inactivation ratio in inaccessible tissues. J. Med. Genet..

[23] Weaving LS (2003). Effects ofMECP2 mutation type, location and X-inactivation in modulating Rett syndrome phenotype. Am. J. Med. Genet..

[24] Clerc P, Avner P (2006). Random X-chromosome inactivation: skewing lessons for mice and men. Curr. Opin. Genet. Dev..

[25] Peeters SB, Yang C, Brown CJ (2016). Have humans lost control: The elusive X-controlling element. Semin. Cell Dev. Biol..

[26] Plenge RM, Stevenson RA, Lubs HA, Schwartz CE, Willard HF (2002). Report Skewed X-Chromosome Inactivation Is a Common Feature of X-Linked Mental Retardation Disorders. Am. J. Hum. Genet.

[27] Monrós E (2001). Rett syndrome in Spain: mutation analysis and clinical correlations. Brain Dev..

[28] Gibson JH, Williamson SL, Arbuckle S, Christodoulou J (2005). X chromosome inactivation patterns in brain in Rett syndrome: Implications for the disease phenotype. Brain Dev..

[29] Gale RE, Wheadon H, Boulos P, Linch DC (1994). Tissue specificity of X-chromosome inactivation patterns. Blood.

[30] Leonard H, Cobb S, Downs J (2016). Clinical and biological progress over 50 years in Rett syndrome. Nat. Rev. Neurol..

[31] Shahbazian MD, Sun Y, Zoghbi HY (2002). Balanced X chromosome inactivation patterns in the Rett syndrome brain. Am. J. Med. Genet..

[32] Swierczek SI (2012). Methylation of AR locus does not always reflect X chromosome inactivation state. Blood.

[33] Lake B. B., Ai R., Kaeser G. E., Salathia N. S., Yung Y. C., Liu R., Wildberg A., Gao D., Fung H.-L., Chen S., Vijayaraghavan R., Wong J., Chen A., Sheng X., Kaper F., Shen R., Ronaghi M., Fan J.-B., Wang W., Chun J., Zhang K. (2016). Neuronal subtypes and diversity revealed by single-nucleus RNA sequencing of the human brain. Science.

[34] Carlson, R. V., Boyd, K. M. & Webb, D. J. The revision of the Declaration of Helsinki: Past, present and future. Vol. 57, British Journal of Clinical Pharmacology. p. 695–713 (2004). 10.1111/j.1365-2125.2004.02103.x PMC1884510 15151515

[35] Cutler Allen R, Zoghbi HY, Annemarie Moseley IB, Rosenblatt HM, Belmont JW (1992). Methylation of Hpall and Hhal Sites Near the Polymorphic CAG Repeat in the Human Androgen-Receptor Gene Correlates with X Chromosome Inactivation. Am. J. Hum. Genet.

[36] Liu J (2012). An improved allele-specific PCR primer design method for SNP marker analysis and its application. Plant Methods.

[37] Untergasser A (2012). Primer3-new capabilities and interfaces. Nucleic Acids Res..

[38] Koressaar T, Remm M (2007). Enhancements and modifications of primer design program Primer3. Bioinformatics.

