# Supplementary material for: X chromosome inactivation does not necessarily determine the severity of the phenotype in Rett syndrome patients
Source: Sci Rep. 2019 Aug 19;9:11983. doi: 10.1038/s41598-019-48385-w (PMC6700087; doi:10.1038/s41598-019-48385-w)
Supplement: Supplementary file 1 — X chromosome inactivation does not necessarily determine the severity of the phenotype in Rett syndrome patients [file 41598_2019_48385_MOESM1_ESM.docx]

**X chromosome inactivation does not necessarily determine the severity of the phenotype in Rett syndrome patients**

Clara Xiol^1^, Silvia Vidal^1^, Ainhoa Pascual-Alonso^1^, Laura Blasco^1^, Núria Brandi^2^, Paola Pacheco^1^, Edgar Gerotina^1^, Mar O'Callaghan^5^, Mercè Pineda^3^, Rett Working Group & Judith Armstrong*^1,3,4^

^1^Molecular and Genetics Medicine Section, Hospital Sant Joan de Déu, Barcelona, Spain

^2^Facultat de Medicina, Universitat de Barcelona, Barcelona, Spain

^3^Institut de Recerca Pediàtrica, Hospital Sant Joan de Déu, Barcelona, Spain

^4^CIBER-ER (Biomedical Network Research Center for Rare Diseases), Instituto de Salud Carlos III, Madrid, Spain

^5^Neurology Service, Hospital Sant Joan de Déu, Barcelona, Spain

**Corresponding author**:

J. Armstrong,

Molecular and Genetics Medicine Section

**Hospital Sant Joan de Déu**

Pg. Sant Joan de Déu 2, planta 0

08950 Esplugues de Llobregat, Barcelona

T. + 34 93 600 9451

F. + 34 93 600 9760

[jarmstrong@sjdhospitalbarcelona.org](mailto:jarmstrong@hsjdbcn.org)

| **Patient Number** | **XCI-AR** | **XCI-AS** | | **Global Score** |
| --- | --- | --- | --- | --- |
|  |  | **WT** | **Mut** |  |
| **Patients with c.455C>G (p.P152R) mutation** | | | | X̅=11.6 (SD=3.782) |
| P1 | n.i. | 53.5 | 46.5 | 8 |
| P2 | 60:40 | 62 | 38 | 13 |
| P3 | 61:39 | 59.5 | 40.5 | 8 |
| P4 | 74:26 | 48.5 | 51.5 | 17 |
| P5 | 68:32 | 59.5 | 40.5 | NA |
| P6 | 72:28 | 34 | 66 | 12 |
| **Patients with c.473C>T (p.T158M) mutation** | | | | X̅=13.22 (SD=3.489) |
| P7 | 72:28 | 37 | 63 | 10 |
| P8 | 54:46 | 66.5 | 33.5 | 16 |
| P9a | 58:42 | 60.5 | 39.5 | 12 |
| P9b | 56:44 | 36 | 64 |  |
| P10 | 54:46 | 37.5 | 62.5 | 12 |
| P11 | 55:45 | 52.5 | 47.5 | 12 |
| P12 | 55:45 | 65 | 35 | 11 |
| P13 | 62:38 | 35.5 | 64.5 | 16 |
| P14 | 65:35 | 36 | 64 | 9 |
| P15 | 61:39 | 44 | 56 | 11 |
| P16 | 53:47 | 54 | 46 | 16 |
| P17 | 56:44 | 42 | 58 | 18 |
| P18 | n.i. | 41.5 | 58.5 | 16 |
| P19 | 67:33 | 35.5 | 64.5 | 11 |
| P20 | n.i. | 57 | 43 | NA |
| P21 | 62:38 | 36.5 | 63.5 | 15 |
| P22 | 79:21 | 41 | 59 | 19 |
| P23 | 71:29 | 46 | 54 | NA |
| P24 | 59:41 | 63 | 37 | 14 |
| P25 | 51:49 | 31.5 | 68.5 | 19 |
| P26 | 63:37 | 41.5 | 58.5 | 9 |
| P27 | NA | NA | NA | 13 |
| P28 | NA | NA | NA | 17 |
| P29 | 73:27 | 59 | 41 | 6 |
| P30 | 71:29 | 51 | 49 | 14 |
| P31 | 62:38 | 48.5 | 51.5 | NA |
| P32 | 63:37 | 39.5 | 60.5 | 10 |
| P33 | n.i. | 48 | 52 | 7 |
| P34 | 68:32 | 56 | 44 | 19 |
| P35 | n.i. | NA | NA | 16 |
| P36 | 71:29 | 41 | 59 | 10 |
| P37 | 71:29 | 50.5 | 49.5 | 12 |
| P38 | 55:45 | 37.5 | 62.5 | NA |
| P39 | 77:23 | NA | NA | NA |
| P40 | 54:46 | 45 | 55 | 14 |
| P41 | 64:36 | 52 | 48 | NA |
| P42 | NA | NA | NA | 13 |
| **Patients with c.502C>T (p.R168X) mutation** | | | | X̅=13.12 (3.361) |
| P43 | 66:34 | 46 | 54 | 13 |
| P44 | 64:36 | 44 | 56 | 17 |
| P45 | 65:35 | 60.5 | 39.5 | 11 |
| P46 | 69:31 | 32 | 68 | 10 |
| P47 | n.i. | 81.5 | 18.5 | 13 |
| P48 | 70:30 | 66.5 | 33.5 | 7 |
| P49 | 51:49 | 56 | 44 | 12 |
| P50 | 68:32 | 48 | 52 | 19 |
| P51 | NA | NA | NA | 19 |
| P52 | 64:36 | 49.5 | 50.5 | 16 |
| P53 | 63:37 | 40 | 60 | 7 |
| P54 | NA | NA | NA | 17 |
| P55 | 59:41 | 38.5 | 61.5 | 14 |
| P56 | 56:44 | 43 | 57 | 13 |
| P57 | n.i. | 61 | 39 | 10 |
| P58 | 64:36 | 38.5 | 61.5 | 11 |
| P59 | n.i. | 59 | 41 | 14 |
| P60 | 84:16 | 28 | 72 | 16 |
| P61 | 58:44 | 35 | 65 | 13 |
| P62 | NA | NA | NA | 12 |
| P63 | 70:30 | 54 | 46 | 17 |
| P64 | NA | NA | NA | 17 |
| P65 | 65:35 | 56.5 | 43.5 | 7 |
| P66 | 76:24 | 43.5 | 56.5 | 10 |
| P67 | 58:42 | 45 | 55 | NA |
| P68 | 75:25 | 15.5 | 84.5 | NA |
| P69 | n.i. | 46 | 54 | 10 |
| P70 | 85:15 | 35 | 65 | NA |
| P71 | 60:40 | 41.5 | 58.5 | 12 |
| P72 | n.i. | 46.5 | 53.5 | 11 |
| P73 | 59:41 | 42 | 58 | 18 |
| P74 | 81:19 | 55.5 | 44.5 | NA |
| P75 | 54:46 | 50 | 50 | 17 |
| P76 | NA | NA | NA | 14 |
| P77 | NA | NA | NA | 7 |
| P78 | NA | NA | NA | 12 |
| P79 | NA | NA | NA | 11 |
| P80 | NA | NA | NA | 13 |
| **Patients with c.674C>G (p.P225R) mutation** | | | |  |
| P81 | 57:43 | 54 | 46 | NA |
| P82 | 67:33 | 55.5 | 44.5 | 11 |
| **Patients with c.763C>T (p.R255X) mutation** | | | | X̅=15.21 (SD=3.213) |
| P83 | 85:15 | 57 | 43 | NA |
| P84 | 87:13 | 55.5 | 44.5 | 13 |
| P85 | 80:20 | 28 | 72 | 14 |
| P86 | 78,5:21,5 | 71.5 | 28.5 | 14 |
| P87 | 76,5:23,5 | 63.5 | 36.5 | 9 |
| P88 | 78:22 | 53.5 | 46.5 | 17 |
| P89 | 75:25 | 25 | 75 | 18 |
| P90 | 75:25 | 63 | 37 | 17 |
| P91 | NA | NA | NA | 10 |
| P92 | 73:27 | 33 | 67 | 18 |
| P93 | 71:29 | 62 | 38 | 13 |
| P94 | 65:35 | 32 | 68 | 19 |
| P95 | 65:35 | 52 | 48 | 17 |
| P96 | 65:35 | 51 | 49 | 18 |
| P97 | 64:36 | 43 | 57 | 14 |
| P98 | 64:36 | 52 | 48 | NA |
| P99 | 63:37 | 51 | 49 | 11 |
| P100 | 60:40 | 68 | 32 | 12 |
| P101 | 59:41 | 60 | 40 | 14 |
| P102 | 57:43 | 71.5 | 28.5 | 18 |
| P103 | 55:45 | 57 | 43 | 15 |
| P104 | 51:49 | 60 | 40 | 17 |
| P105 | n.i. | 45 | 55 | 17 |
| P106 | 62:38 | 62 | 38 | 17 |
| P107 | 87:13 | 68 | 32 | 11 |
| P108 | 75:25 | 68 | 32 | 17 |
| P109 | 73:27 | 58 | 42 | 20 |
| P110 | 68:32 | 60 | 40 | 13 |
| P111 | 65:35 | 49 | 51 | 15 |
| P112 | 64:36 | 45 | 55 | NA |
| P113 | 64:36 | 56 | 44 | NA |
| P114 | 60:40 | 50 | 50 | 15 |
| P115 | 58:42 | 53 | 47 | NA |
| P116 | NA | NA | NA | 18 |
| P117 | 52:48 | 40 | 60 | NA |
| P118 | 51:49 | 62 | 38 | NA |
| P119 | n.i. | 34 | 66 | 19 |
| P120 | 58:42 | 48 | 52 | NA |
| P121 | NA | NA | NA | 20 |
| P122 | NA | NA | NA | 17 |
| P123 | NA | NA | NA | 10 |
| P124 | NA | NA | NA | 19 |
| P125 | NA | NA | NA | 10 |
| P126 | NA | NA | NA | 16 |
| P127 | NA | NA | NA | 10 |
| P128 | NA | NA | NA | 19 |
| P129 | NA | NA | NA | 12 |
| **Patients with c.806delG (p.G269fs) mutation** | | | | X̅=14.29 (SD=4.112) |
| P130 | 77:23 | 51.5 | 48.5 | 8 |
| P131 | 76:24 | 36.5 | 64.5 | NA |
| P132 | 76:24 | 65 | 35 | 14 |
| P133 | n.i. | 52.5 | 47.5 | 20 |
| P134 | n.i. | 55.5 | 44.5 | 16 |
| P135 | 59:41 | 43.5 | 56.5 | 10 |
| P136 | 68:32 | 59 | 41 | 15 |
| P137 | 54:46 | 41 | 59 | 17 |
| P138 | 77:23 | 52 | 48 | NA |
| P139 | 82:18 | 58 | 42 | NA |
| P140 | 68:32 | 57.5 | 42.5 | NA |
| P141 | NA | NA | NA | NA |
| P142 | NA | NA | NA | NA |
| **Patients with c.808C>T (p.R270X) mutation** | | | | X̅=14.69 (SD=3.846) |
| P143 | 97:3 | 16 | 84 | 18 |
| P144 | 84:16 | 21 | 79 | NA |
| P145 | 81:19 | 30 | 70 | 9 |
| P146 | 80:20 | 73 | 27 | 13 |
| P147 | 73:27 | 62 | 38 | 19 |
| P148 | 71.5:28.5 | 33 | 67 | 11 |
| P149 | 72.5:27.5 | 58 | 42 | 11 |
| P150 | 69:31 | 59 | 41 | 17 |
| P151 | 69:31 | 48 | 52 | 22 |
| P152 | 65:35 | 53 | 47 | 17 |
| P153 | 64:36 | 61 | 39 | 8 |
| P154 | 68:32 | 37 | 63 | 16 |
| P155 | NA | NA | NA | 17 |
| P156 | 54:46 | 56 | 44 | 19 |
| P157 | 53:47 | 39 | 61 | 16 |
| P158 | 53:47 | 56 | 44 | 16 |
| P159 | 53:47 | 52 | 48 | 16 |
| P160 | 53:47 | 36 | 64 | 18 |
| P161 | 52:48 | 57 | 43 | 17 |
| P162 | 62:38 | 54.5 | 45.5 | NA |
| P163 | 58:42 | 48 | 52 | 21 |
| P164 | NA | NA | NA | 13 |
| P165 | NA | NA | NA | 9 |
| P166 | NA | NA | NA | 12 |
| P167 | NA | NA | NA | 16 |
| P168 | NA | NA | NA | 11 |
| P169 | NA | NA | NA | 14 |
| P170 | NA | NA | NA | 10 |
| P171 | NA | NA | NA | 9 |
| P172 | NA | NA | NA | 18 |
| P173 | NA | NA | NA | 13 |
| **Patients with c.880C>T (p.R294X) mutation** | | | | X̅=10.46 (SD=2.993) |
| P174 | 71:29 | 48 | 52 | 7 |
| P175 | 59:41 | 55 | 45 | 11 |
| P176 | 58:42 | 40 | 60 | 12 |
| P177 | 67:33 | 51 | 49 | 16 |
| P178 | 59:41 | 37.5 | 62.5 | 13 |
| P179 | 57:43 | 53.5 | 46.5 | NA |
| P180 | 75:25 | 54 | 46 | 13 |
| P181 | 56:44 | NA | NA | 7 |
| P182 | 55:45 | 65 | 35 | NA |
| P183 | 54:46 | 51 | 49 | 10 |
| P184 | 55:45 | 52.5 | 47.5 | NA |
| P185 | 62:38 | NA | NA | NA |
| P186 | 53:47 | NA | NA | 12 |
| P187 | 56:44 | NA | NA | NA |
| P188 | 53:47 | 44 | 56 | 12 |
| P189 | 62:38 | 46 | 54 | NA |
| P190 | 59:41 | NA | NA | 6 |
| P191 | 89:11 | 49 | 51 | NA |
| P192 | 67:33 | 42 | 58 | 8 |
| P193 | 75:25 | 34 | 66 | NA |
| P194 | NA | NA | NA | 9 |
| **Patients with c.916C>T (p.R306C) mutation** | | | | X̅=11.18 (SD=3.065) |
| P195 | 89:11 | 59.5 | 40.5 | 9 |
| P196a | 70.5:29.5 | 60 | 40 | 14 |
| P196b | 45:55 | 63 | 37 |  |
| P197 | 70:30 | 45 | 55 | 9 |
| P198 | 63:37 | 63 | 37 | 11 |
| P199 | 62:38 | 76 | 24 | 12 |
| P200 | 59:41 | 49 | 51 | 16 |
| P201 | 59:41 | 56 | 44 | NA |
| P202 | 59:41 | 63 | 37 | 10 |
| P203 | 58:42 | 66 | 34 | 7 |
| P204 | 56.5:43.5 | 47 | 53 | 16 |
| P205 | 61:39 | 31 | 69 | 14 |
| P206 | 60:40 | 32 | 68 | 12 |
| P207 | 64:36 | 38 | 62 | 16 |
| P208 | 70:30 | 25 | 75 | NA |
| P209 | n.i. | 38 | 62 | NA |
| P210 | NA | NA | NA | 12 |
| P211 | NA | NA | NA | 10 |
| P212 | NA | NA | NA | 7 |
| P213 | NA | NA | NA | 7 |
| P214 | NA | NA | NA | 11 |
| P215 | NA | NA | NA | 11 |
| P216 | NA | NA | NA | 13 |
| P217 | NA | NA | NA | 11 |
| P218 | NA | NA | NA | 5 |
| P219 | NA | NA | NA | 13 |
| **Patients with deletions in *MECP2*** | | |  |  |
| P220 | 88:12 | 6.7 | 93.3 | NA |
| P221 | 74:26 | 51.3 | 48.7 | 15 |

**Table S1**. **Patient data.** Data of patients with the c.455C>G mutation (n=6), c.473C>T mutation (n=36), c.502C>T mutation (n=38), c.674C>G mutation (n=2), c.763C>T mutation (n=47), c.806delG mutation (n=13), c.808C>T mutation (n=31), c.880C>T (n=21), c.916C>T mutation (n=25) and patients with a deletion in *MECP2* (n=2). The XCI-AR column shows the results of the AR XCI assay (percentage of inactivation of each allele). The XCI-AS WT and Mut columns show the results of the allele-specific XCI assay (percentage of inactivation of each allele, mean of two replicates n=2 or three replicates n=3 in the cases of the deletions). The Global Score column shows the average (X̅) score and its standard deviation (SD) in brackets for the patients of our cohort with each mutation. n.i. = polymorphism noninformative for the assay. NA = data not available.

| **Locus** | **Forward Primer** | | **Reverse Primer** | |
| --- | --- | --- | --- | --- |
| *AR* | TCCAGAATCTGTTCCAGAGCGTGC | | *GCTGTGAAGGTTGCTGTTCCTCAT | |
| *MECP2* c.455C>G (p.Pro152Arg) | WT | GCGACACATCCCTGGAC**TC** | *AGCTTCCCAGGACTTTTCTCC | |
|  | Mut | GCGACACATCCCTGGAC**TG** |  |  |
| *MECP2* c.473C>T (p.Thr158Met) | WT | GGACCCTAATGATTTTGACTT**T**A**C** |  |  |
|  | Mut | GGACCCTAATGATTTTGACTT**T**A**T** |  |  |
| *MECP2* c.502C>T (p.Arg168*) | *TCGAAAAGGTAGGCGACACATC | | WT | CTTAGGTGGTTTCTGCTC**C**C**G** |
|  |  |  | Mut | CTTAGGTGGTTTCTGCTC**C**C**A** |
| *MECP2* c.674C>G (p.ProP225Leu) |  |  | WT | CCCCTGGCGAAGTTTGAAA**GG** |
|  |  |  | Mut | CCCCTGGCGAAGTTTGAAA**GC** |
| *MECP2* c.763C>T (p.Arg255*) | WT | AAACGCCCCGGCAGGA**G**G**C** | *AGTCCTTTCCCGCTCTTCTC | |
|  | Mut | AAACGCCCCGGCAGGA**G**G**T** |  |  |
| *MECP2* c.806delG (p.Gly269fs) | WT | ATTCCCAAGAAACGGG**G**CCG |  |  |
|  | Mut | ATTCCCAAGAAACGGGCCGA |  |  |
| *MECP2* c.808C>T (p.Arg270*) | WT | CATTCCCAAGAAACGGG**A**C**C** |  |  |
|  | Mut | CATTCCCAAGAAACGGG**A**C**T** |  |  |
| *MECP2* c.880C>T (p.Arg294*) | WT | *TCGAAAAGGTAGGCGACACATC | GGTCTCCTGCACAGA**A**C**G** | |
|  | Mut |  | GGTCTCCTGCACAGA**A**C**A** | |
| *MECP2* c.916C>T (p.Arg306Cys) | WT | ACCGTACTCCCCATCAAGA**G**G**C** | *TCTGAGTGGTGGTGATGGTG | |
|  | Mut | ACCGTACTCCCCATCAAGA**G**G**T** |  |  |
| MECP2 c.27-10677_1192del (**deletion 1**; exon 3 and 4, 12599bp) | WT | acattcaggttggcttggtc | *CTCTGGGCATCTTCTCCTCTT | |
|  | Mut | aaaaataaaatgtgcaattcagtgtc |  |  |
| *MECP2* c.887_10015+18460del (**deletion 2**; exon 4 and *IRAK1*, 27589bp) | WT | TCTGAGTGGTGGTGATGGTG | *CAGGCCATTCCCAAGAAAC | |
|  | Mut | CTCAGCTGGAAGGGAAAATG |  |  |
| *MECP2* c.763C>T (p.Arg255*) Sanger Sequencing | GGACCCTAATGATTTTGACTTTAC | | GGTCTCCTGCACAGAACG | |
|  |  |  |  |  |

**Table S2. Primer sequences.** Primer sequences to amplify each locus. Bold yellow formatting indicates the mutated nucleotides and bold red formatting indicates the mismatch introduced for primer specificity. The bold green formatting indicates the nucleotide only present in the WT allele and not in the mutated allele with the G deletion. * Indicates the FAM^TM^ label at the 5’ end of the primer to allow detection in fragment analysis. Mutation nomenclature is referenced to *MECP2* NM_004992.3.

|  | **PCR Mix 1** | | **PCR Mix 2** | | **PCR Mix 3** | |
| --- | --- | --- | --- | --- | --- | --- |
|  | **PCR Mix** | **Final concentration** | **PCR Mix** | **Final concentration** | **PCR Mix** | **Final concentration** |
| H_2_O | 19.3μL | - | 18.25μL | - | 17.25μL | - |
| Buffer | 2.5μL | 1x (2.5mM MgCl_2_) | 2.5μL | 1x (2.5mM MgCl_2_) | 2.5μL + 1μL MgCl_2_ | 1x (1mM MgCl_2_) |
| dNTPs | 0.5μL | 0.2mM | 1μL | 0.4mM | 1μL | 0.4mM |
| Primers | 0.5μL + 0.5μL | 0.2μM | 1μL + 1μL | 0.4μM | 1μL +1μL | 0.4μM |
| HiFi Taq Polymerase | 0.2μL | 0.028U/μL | 0.25μL | 0.035U/μL | 0.25μL | 0.035U/μL |
| Total Mix | 23.5μL |  | 24μL |  | 24μL |  |
| DNA | 1.5μL | 1.2ng/μL | 1μL | 0.8ng/μL | 1μL | 0.8ng/μL |
| Total | 25μL |  | 25μL |  | 25μL |  |

**Table S3. Amplification conditions.** Amplification conditions for all assays. PCR Mix 1 was used for the AR, c.455C>G, c.473C>T, c.502C>T c.763C>T, c.808C>T and c.916C>T assays; PCR Mix 2 was used for the c.674C>G, c.880C>T assays and the deletion assays; PCR Mix 3 was used for the c.806delG assay. The buffer used in PCR mixes 1 and 2 already contains MgCl_2_, whereas in PCR Mix 3 the MgCl_2_ is added separately.

| **Amplification**  **Program** | | |
| --- | --- | --- |
|  |  |  |
| 96ºC | 5min |  |
| 96ºC | 45sec | C cycles |
| TºC | 45sec |  |
| 72ºC | 45sec |  |
| 72ºC | 7min |  |

**Table S4. Amplification program.** Annealing temperature (T) varied between primer pairs: AR 60ºC, c.455C>G 58ºC, c.473C>T 61ºC, c.502C>T 61ºC, c.674C>T 60ºC, c.763C>T 60ºC, c.806delG 61ºC, c.808C>T 58ºC, c.880C>T 56ºC, c.916C>T 60ºC and deletion assays 59ºC. The number of cycles (C) also varied between the AR assay (25 cycles) and the allele-specific assays (30 cycles).

| **Primer/Probe** | **Sequence** |
| --- | --- |
| *MECP2* Forward Primer | CCAGGTCATGGTGATCAAACG |
| *MECP2* Reverse Primer | AGACTCCTTCACGGCTTTCT |
| *MECP2* WT Probe | CAGCTTTTC**G**CTTCCTG |
| *MECP2* Mut Probe | CAGCTTTTC**A**CTTCCTG |

**Table S5. TaqMan® probe and primer sequences.** Primer and probe sequences for TaqMan^TM^ qPCR assay. Bold colored formatting indicates the different nucleotides in the TaqMan^TM^ probes.
